# Premature ventricular contraction originating from a papillary muscle‐chordae transition inside the left ventricle

**DOI:** 10.1002/joa3.12765

**Published:** 2022-08-08

**Authors:** Tsukasa Naganuma, Hitoshi Mori, Kenta Tsutsui, Haruka Yamazaki, Ritsushi Kato

**Affiliations:** ^1^ Department of Cardiology Saitama Medical University International Medical Center Saitama Japan; ^2^ Department of Medical Engineer Saitama Medical University International Medical Center Saitama Japan

**Keywords:** 3D mapping, catheter ablation, papillary muscle, premature ventricular contraction

Premature ventricular contractions (PVCs) originate from left ventricle (LV) papillary muscle (PM) in 5%–10% of idiopathic PVCs and 14% of those origins are located in the basal third of the PM.^1^, ^2^ Treatment of such PM‐related PVCs is challenging; the recurrence rate following percutaneous radiofrequency catheter ablation (RFCA) is higher than that for other types of idiopathic PVCs due to the vigorous movement and tissue thickness, i.e., evidence remains scarce on the treatment of PM‐related PVCs; several reports have demonstrated the usefulness of intracardiac echocardiography and pace mapping.^3^, ^4^ Here, we report a unique form of PM‐related PVCs that emerged from PM‐tendon transitions, which were successfully treated with RFCA. A combination of intracardiac echocardiography and PASO® Module mapping with a DecaNav catheter critically contributed to the success.

A 55‐year‐old woman was referred to our hospital for RFCA of frequent PVCs with a QRS width of 220 ms, complete right bundle branch block, and a superior axis (Figure 1A,B). She had a past medical history of a posterolateral myocardial infarction and was treated for chronic heart failure since then. A Holter electrocardiography revealed frequent monomorphic PVCs at 45 000 beats/day. The LV ejection fraction was further reduced to 35% from 50%, which was already reduced before the PVCs became frequent. Carvedilol 20 mg failed to suppress the PVC burden.

**FIGURE 1:**
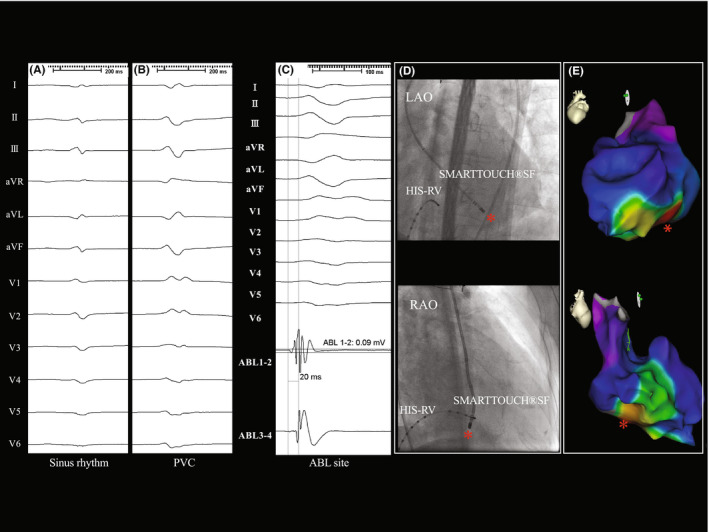
(A) Surface electrograms of sinus rhythm. (B) The clinical PVC. (C) At the ablation site, a pre‐potential that preceded the QRS by 20 ms was recorded during a spontaneous, clinical PVC. The pace‐map score at that point was also highest at 0.87 as compared to the other points. The location is shown as the red star in (E). (D) Fluoroscopic images. A red star indicates the location at which the electrogram in (C) and the earliest activation site in (F) were recorded. LAO, left anterior oblique, RAO, right anterior oblique. (E) Three‐dimensional anatomic maps of the LV. The red star indicates the ablation point.

In the first attempt at catheter ablation, a 3D mapping system (CARTO3®, Biosense Webster) and open‐irrigated tip radiofrequency catheter (SMARTTOUCH®SF, Biosense Webster) were used to create an activation map, which exhibited a centrifugal propagation pattern from the inferior LV wall near the posterior PM (Figure 1E). The local potential at the earliest activation site preceded the QRS onset of the surface 12‐lead ECG by 20 ms at 0.09 mV (Figure 1C). The PVCs were mechanically suppressed when the ablation catheter made strong contact with the earliest activation site (Figure 1D,E). Following transient reactive firings, ablation with 30 W apparently abolished the PVC. The procedure was concluded after a 30‐min waiting period.

Because the same morphology of PVCs recurred 3 months after the first session (Figure 2A,B), a second ablation session was performed. To better determine the source of the origin, a linear decapolar mapping catheter (DecaNav, Biosense Webster) was used. In addition to a conventional activation map, we created a “pace‐map” (Figure 2C–F, dubbed “PASO® mapping”), in which the PASO® score (calculated by the PASO® Module and ranged from 0.00 to 1.00 with 1.00 being the highest, CARTO 3 system, Biosense Webster) for each point was displayed in a color, to help visualize the spatial distribution of the PASO® scores. The scores tended to be high in the area close to the posterior PM (Figure 2C–F). We discovered that the score was markedly high at 0.97 at an inner point floating in the LV chamber (Figure 2E, “E” in Figure 2F). An ultrasound catheter with an intracardiac magnetic sensor (SOUND STAR®, Biosense Webster) clearly demonstrated that the earliest point was exactly at the junction between the posterior PM and chordae tendineae (Figure 3A,B, Video S1). The onset of the local potential at that point preceded that of the surface 12‐lead ECG QRS by 43 ms with 0.05 mV (Figure 3C). While monitoring the catheter contact with intracardiac echocardiography, RF energy (30 W) was delivered at that point with an average contact force of 5–8 g (Figure 3B,D). Immediately after the start of the energy delivery, the PVC disappeared. Neither spontaneous nor induced PVCs were observed with or without an intravenous infusion of isoproterenol during a 30‐min waiting time. The procedure was concluded. Unlike the first attempt, to date, no PVCs have recurred.

**FIGURE 2:**
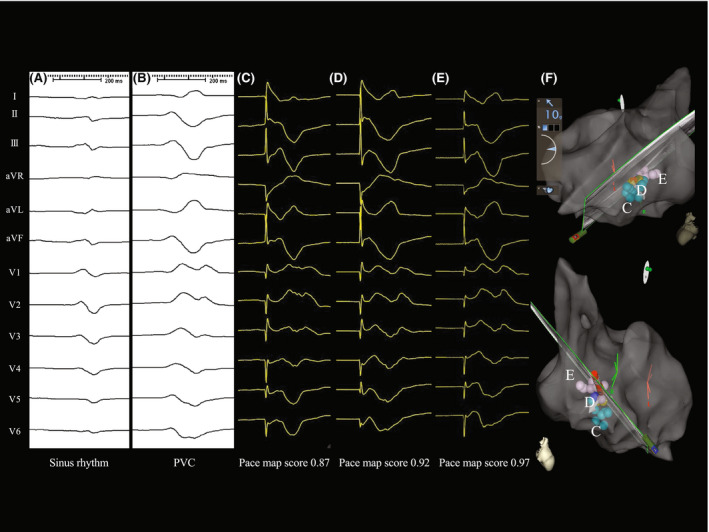
(A) Surface electrograms of sinus rhythm. (B) The clinical PVC. The waveform was identical to that in the 1st session (Figure 1(B)). (C–E) Illustrative examples of PASO® maps that were recorded along the posterior papillary muscle. The locations of (C–E) are annotated in (F). (C) The pace‐map score was 0.87 at the base of the posterior papillary muscle. (D) The pace‐map score was 0.92 at the mid‐posterior papillary muscle. (E) The pace‐map score was 0.97 at the papillary muscle‐chordae tendineae transition. (F) An electroanatomical map located the highest PASO® score of 0.97 (that in E, annotated as “E”). Note that, because these points were on the posterior papillary muscle, they were distant from the LV free wall (this is in contrast to Figure 1(F)).

**FIGURE 3:**
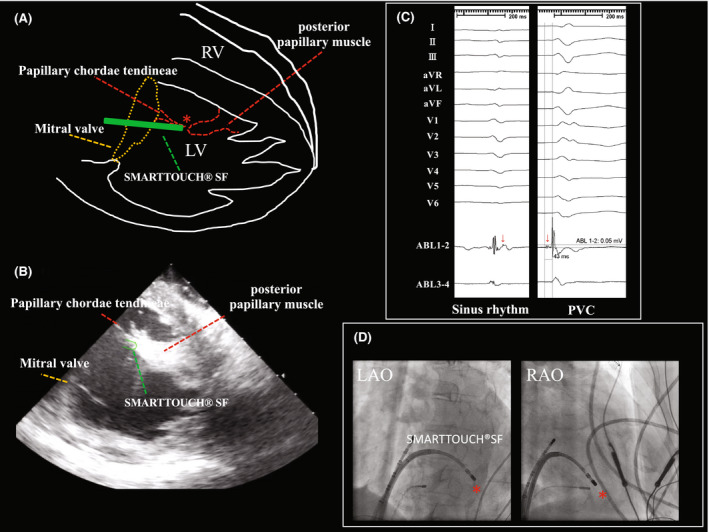
(A) The intraventricular schema: the point where the SMARTTOUCH®SF tip contacts the tissue, and the surrounding left ventricular anatomy are highlighted in colors (ablation catheter, red; papillary muscle, red; and mitral valve, yellow). The red star indicates where the electrogram shown in (C) was recorded. (B) The intracardiac echocardiogram corresponding to the schema in (A). (C) The high score point in the PASO® map had a late potential during sinus rhythm (arrow in the left panel) and a pre‐potential that preceded the QRS by 43 ms in a spontaneous PVC (arrow in the right panel). (D) The catheter placement in the fluoroscopic images. The red stars in both panels correspond to the star shown in (A), i.e., where the highest PASO® score and electrogram in (C) were recorded.

To the best of our knowledge, this is the first report of treating PVCs emerging from the transition between an LV PM and chordae tendineae with the PASO® Module by using a DecaNav catheter. In general, ablation of PM‐related PVCs can be challenging. Constant movement, complex and elusive intracardiac structures and the thickness of the tissue all make it difficult to obtain a precise activation map and adequate contact force, which are both crucial for a curative RF ablation. As previously suggested in typical PM‐related PVCs, the stability of the catheter contact and catheter navigation by intracardiac echocardiography also critically contributed to the success of the present case.^5^

A retrospective analysis indicated the possibility that the point‐by‐point activation map during the 1st session misleadingly projected captured electroanatomical information from the LV tissue including the free wall and PM onto the free wall (not PM, Figure 1F), deceiving us into targeting an inaccurate site, culminating in the failure of the ablation. This interpretation was reinforced by the finding during the 2nd session in which the highest PASO® score was obtained from a floating point within the LV (Figure 2C–F), which was then confirmed to be a PM‐tendon junction by intracardiac echocardiography (Figure 3A,B).

We also considered that the PASO® map using a DecaNav catheter was a major contributing factor to the success in the 2nd session. By sequentially pacing the LV muscle from the distal to proximal pairs of electrodes along the catheter shaft, our technique of using a DecaNav catheter could create pace‐map scores at different locations including ones distant from the LV free wall, such as the PM, and it was useful for discovering the true origin of the PVCs at the PM‐chordae tendineae junction. Furthermore, the electrode width and spacing of the DecaNav catheter were 1 and 1 mm (edge to edge distance of 3.0 mm), respectively. While that of the STSF catheter was 3.5 mm (distal tip)/1.0 mm (2nd ring) and 1 mm (edge to edge distance of 5.5 mm). The small electrode width and spacing contributed to capturing the local ventricle muscle and obtaining a good PASO® score in the 2nd session.

We experienced an atypical manifestation of PM‐related PVCs originating from the PM‐chordae tendineae junction. The combination of the DecaNav catheter, intracardiac echocardiography, and PASO® score map was useful for targeting the PVC.

## FUNDING INFORMATION

None.

## CONFLICT OF INTEREST

The authors declare no conflict of interest for this article.

## ETHICS STATEMENT

Not applicable.

## CONSENT FOR PUBLICATION

Patient consent for publication was obtained.

## CLINICAL TRIAL REGISTRATION

Not applicable.

## Supporting information


Video S1
